# Longitudinal atherosclerotic changes after radio(chemo)therapy of hypopharyngeal carcinoma

**DOI:** 10.1186/s13014-020-01541-3

**Published:** 2020-05-07

**Authors:** Cristoforo Simonetto, Michael Mayinger, Thamer Ahmed, Kai Borm, Pavel Kundrát, Steffi Pigorsch, Jan Christian Kaiser, Stephanie E. Combs

**Affiliations:** 1 Helmholtz Zentrum München GmbH, Deutsches Forschungszentrum für Gesundheit und Umwelt (GmbH), German Research Center for Environmental Health, Institute of Radiation Medicine, Ingolstädter Landstraße 1, D-85764 Neuherberg, Germany; 2 Department of Radiation Oncology, Klinikum rechts der Isar, Technical University of Munich (TUM), Munich, Germany; 3 Department of Radiation Oncology, University Hospital Zurich, University of Zurich, Zurich, Switzerland; 4 Deutsches Konsortium für Translationale Krebsforschung (DKTK), Partnerstandort München, Munich, Germany

**Keywords:** Hypopharyngeal carcinoma, Radiotherapy, Atherosclerosis, Intima media thickness, Plaque, Diabetes mellitus

## Abstract

**Background:**

Radiotherapy treatment of head and neck cancer affects local arteries and increases the risk of stroke. This study aimed at a closer characterization of this damage and its development in time with a longitudinal study set up.

**Methods:**

Male patients treated between 2011 and 2016 for hypopharyngeal carcinoma were identified from the in-house clinical data base. They were included into the study if besides the planning CT at least one additional CT image was available from follow-up (13 patients) or at least two MRI scans (16 patients of which 2 were already included). All patients received radiotherapy, and chemotherapy was administered to 16 patients. The time from the beginning of radiotherapy to the last available image ranged from 2 months to 4.5 years.

For six segments of the carotid arteries, the number and volume of atherosclerotic plaques were determined from the CT scans, and the intima media thickness from the MRI scans. Information on comorbid cardiovascular disease, hypertension and diabetes mellitus was retrieved from medical records.

**Results:**

Total plaque volume rose from 0.25 cm^3^ before to 0.33 cm^3^ after therapy but this was not significant (*p* = 0.26). The mean number of plaques increased from 5.7 to 8.1 (*p* = 0.002), and the intima media thickened from 1.17 mm to 1.35 mm (p = 0.002). However, the mean intima media thickness practically did not change in patients with comorbid diabetes mellitus (*p*-value for homogeneity: 0.03). For patients without diabetes mellitus, dynamics of both plaque number and intima media thickness, was consistent with an increase until about one year after therapy and no further progression thereafter.

**Conclusion:**

Our study confirmed the thickening of artery walls and the increase in the number of plaques. Results imply that definitive radiation damage to the artery walls can be determined not earlier than about one year after radiotherapy and there is no substantial deterioration thereafter. Reasons for the absence of an observable intima media thickening in patients with diabetes are unclear.

## Introduction

Radiotherapy (RT) of the head and neck can lead to vasculopathy of medium- and large-diameter arteries and double the risk for ischemic strokes and transient ischemic attacks [1]. With progress in survival for head and neck cancer patients, side effects of the RT treatment become more important. Moreover, radiation vasculopathy might be relevant for all cancer patients with favorable prognosis, and was therefore investigated recently also in breast cancer patients [2].

Radiation vasculopathy can manifest itself in an increase in plaques, thickening of the intima media, and stenosis. These impairments have been primarily observed in ultrasound examinations [3–5] but can also be seen in CT [6–8], and MRI follow-ups [9, 10].

Carotid stenosis is an established side-effect after local cancer radiotherapy [11]. Only few studies, however, have monitored the atherosclerotic development longitudinally from the onset of RT and including several time points [8, 12]. Therefore, the exact dynamics of the arterial deterioration remains unknown. The aim of the present retrospective study was to investigate the time-dependent development of plaques and intima media thickness in patients treated with RT or a combined treatment with RT and chemotherapy for hypopharyngeal cancer.

## Materials and methods

### Patients

Male patients treated with RT for primary hypopharyngeal carcinoma between 2011 and 2016 were identified from the in-house clinical data base. In total, 28 patients were included, who besides a planning CT either had at least one additional CT from the follow-up (13 patients) or at least two MRIs (16 patients). For two patients, both criteria were fulfilled. In total, 30 CTs and 38 MRIs were used for data analysis. Treatment plans and comorbid conditions were collected from the medical records.

### Imaging analysis

Data from CT and MRI performed during routine follow-ups were analyzed.

Data acquisition was performed on a 3 T MRI scanner (Magnetom Verio, Siemens Healthcare, Erlangen, Germany) with a 32-channel head coil array. T1- weighted 3D magnetization rapid- acquisition gradient echo (MP-RAGE) MRI scans in an axial orientation were included in the data set. MRI data was inspected for image quality.

Vessel thickness in the T1 (repetition time: 687; echo time: 12) was measured using a PACS workstation (Picture Archiving and Communication System, AGFA Healthcare Corp., Greenville, SC, USA) at 3 levels: In the internal carotid artery (ICA) at the most caudal image level also showing the cerebellum, 5 mm cranial to the carotid bifurcation and in the common carotid artery (CCA) at the level of the third cervical vertebrae.

For CT imaging, a kilovoltage CT scan (Somatom Emotion 16, Siemens, Erlangen, Germany or Brilliance 64, Philips Healthcare) was performed with an axial slice thickness of 1–3 mm (Iopromid, Bayer Healthcare, Leverkusen, Germany). Plaques were counted as descriped by Walker et al. [13]. Briefly, the attenuation values were measured by use of a circular or elliptical region of interest. All foci with Hounsfield units (HU) deviating more than > 50 compared to the surrounding tissure were considered and counted as plaque.

### Statistical analysis

To investigate significant changes in the measured parameters before and after RT, Wilcoxon signed-rank tests were performed. If two images after RT were available, an average value was calculated. Potential differences between different subgroups were analyzed by Wilcoxon rank-sum tests. The course of changes was fitted by a constant, a categorical, and a continuous model linear in the time after radiotherapy. Goodness of fit was assessed by the sum of squared residuals *χ*^2^, using the constant model as null hypothesis. *P*-values < 0.05 were assumed as statistically significant. All analyses were performed with MATLAB R2018b.

## Results

Patient and treatment characteristics are summarized in Table 1. Mean duration from the beginning of RT to the last available CT was 414 days (range: 91 to 938 days), and to the last available MRI 544 days (range: 63 to 1667 days).

**Table 1:** Characteristics of patients and treatments

Treatment age [years]	Median: 60, range: 44–78
Radiotherapy intent	18 definitive
	6 adjuvant
	1 palliative
	1 additive
Prescribed dose	3 times 54/55 Gy
	7 times 64 Gy
	17 times 70 Gy
Chemotherapy	16 (13 Cisplatin, 1 Carboplatin, 1 Cis- and Carboplatin, 1 Carboplatin and Paclitaxel)
History of cardiovascular disease	6 (3 coronary artery disease, 3 peripheral artery occlusive disease, 1 atrial fibrillation, 1 aortic stenosis, 1 stroke)
History of hypertension	10
History of diabetes	5

In the planning CTs, preexisting plaques were observed for 77% of patients. In 23% of the patients plaques were observed in the ICA at the level of the cerebellum to the middle of the third cervical vertebra, in 77% in the ICA and CCA from the middle of the third cervical vertebra to the middle of the sixth, and in 15% in the CCA at the cervical vertebral level 6 to the pulmonary apex. The average number of plaques was 0.8 for the ICA at the level of the cerebellum, 4.5 for the bifurcation, and 0.3 for the CCA at the cervical vertebral level 6. The average sizes per plaque for the different sites were 27 mm^3^, 48 mm^3^, and 32 mm^3^. Intima media thickness was determined from MRIs. In the 10 patients with MRI scans prior to RT, the mean intima media thickness before RT was 0.97 mm (interquartile range 0.85–1.1) in the ICA at the level of the cerebellum, 1.25 mm (IQR 1.1–1.35) at the bifurcation, and 1.28 mm (IQR 1.0–1.35) in the CCA at the cervical vertebral level 6.

On average, the number of plaques increased from 5.7 (IQR 1–10.5) before RT to 8.1 (IQR 2–13; *p*-value for difference 0.002). The increase of the average total plaque volume from 249 mm^3^ (IQR 7–597) to 326 mm^3^ (IQR 8–540) was, however, not significant (*p* = 0.26). The intima media thickness increased from 1.17 mm (IQR 0.97–1.28) to 1.35 mm (IQR 1.16–1.52; *p* = 0.002). Results before RT and the observed changes are listed in Table 2 for all patients included as well as several patient subgroups

**Table 2:** Increase in plaque number, plaque volume and intima media thickness for all patients included and for different subgroups. Tests for heterogeneity were applied to examine whether increase during follow-up was identical for two alternative subgroups

		Plaque number			Plaque volume [mm^3^]			Intima media thickness (IMT) [mm]		
		Before RT	Increase during follow-up	Test for significance	Before RT	Increase during follow-up	Test for significance	Before RT	Increase during follow-up	Test for significance
All patients		5.7	2.4	**p = 0.002**	249	77	p = 0.26	1.17	0.19	**p = 0.002**
Prescribed dose < 70 Gy	yes	6.8	2.3	*p* = 0.06	308	−16	*p* > 0.5	1.23	0.16	*p* = 0.5
	no	4.7	2.4	p = 0.06	198	157	p = 0.13	1.15	0.20	***p*** **= 0.008**
Test for heterogeneity			p > 0.5			*p* = 0.46			p > 0.5	
Chemotherapy	yes	2.7	1.5	*p* = 0.25	108	81	p = 0.13	1.18	0.20	**p = 0.02**
	no	8.3	3.1	**p = 0.02**	370	73	p > 0.5	1.14	0.16	p = 0.25
Test for heterogeneity			*p* = 0.21			*p* = 0.41			p > 0.5	
Cardiovascular disease	yes	13.7	5.0	p = 0.25	709	194	p > 0.5	1.27	0.14	p = 0.5
	no	3.3	1.6	**p = 0.02**	111	47	*p* = 0.19	1.14	0.20	**p = 0.008**
Test for heterogeneity			*p* = 0.20			p > 0.5			p > 0.5	
Hypertension	yes	8.3	3.2	p = 0.25	466	−9	p > 0.5	1.26	0.10	p = 0.06
	no	4.9	2.2	**p = 0.02**	184	103	p = 0.19	1.07	0.27	p = 0.06
Test for heterogeneity			*p* = 0.27			p > 0.5			***p*** **= 0.03**	
Diabetes mellitus	yes	8.0	4.3	p = 0.5	392	−49	p > 0.5	1.31	0.06	p = 0.25
	no	5.3	2.0	**p = 0.008**	223	100	p = 0.13	1.11	0.24	**p = 0.02**
Test for heterogeneity			*p* = 0.10			p > 0.5			**p = 0.03**	
> 5 plaques before RT	yes	12.6	3.9	p = 0.06	633	116	p > 0.5			
	no	1.4	1.4	p = 0.06	9	52	p = 0.13			
Test for heterogeneity			*p* = 0.14			p > 0.5				
IMT > 1.2 mm before RT	yes							1.28	0.18	**p = 0.03**
	no							0.99	0.21	p = 0.13
Test for heterogeneity									p > 0.5	

No significant difference in the increase of plaque numbers was seen between subgroups but a trend for a higher absolute increase with a pre-existing condition (cardiovascular disease, hypertension, diabetes mellitus, or an above-average number of plaques) was observed. The change in intima media thickness (IMT) was significantly lower for patients with hypertension (10 patients) or diabetes mellitus. Here it has to be noted, that all 5 patients with diabetes mellitus also suffered from hypertension. When excluding patients with diabetes mellitus from the analysis, the increase in IMT for patients with vs. without hypertension was no longer significantly different (*p* = 0.38). Therefore, patients with diabetes mellitus were excluded for further analysis.

**Fig. 1:**
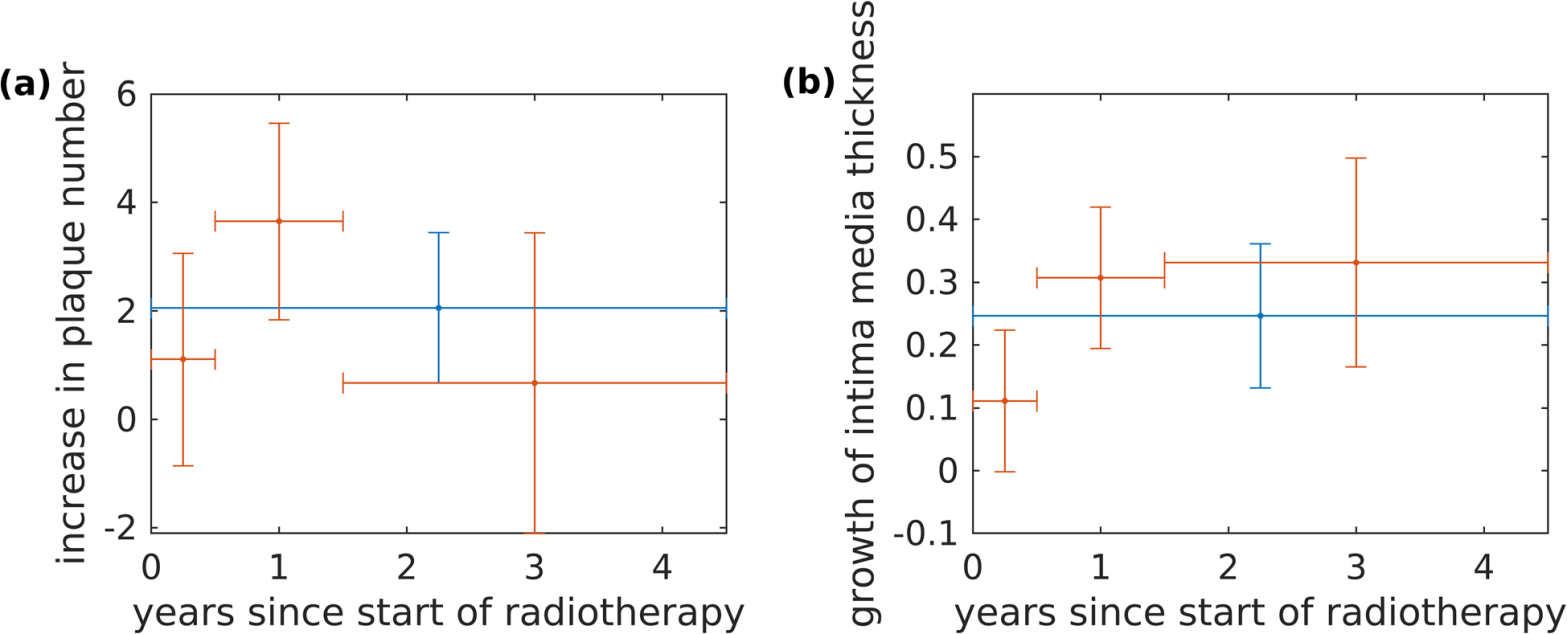
**a** Average (blue) and time-dependent (red) increase of the number of plaques (**a**) and intima media thickness (**b**). Bars represent 90% confidence intervals

The time course of the increase in the number of plaques and the thickening of the intima media were fitted by a categorical model and the results are shown in Fig. 1. For the number of plaques, the categorical model showed a superior goodness of fit but the improvement was not significantly better than the constant model based on a likelihood ratio test (*p* = 0.13). On the other hand, for intima media thickness the categorical model fitted significantly better (*p* = 0.02), implying that thickening takes some time after radiotherapy. To investigate whether a continuous increase would better describe the data, we fitted also a model linear in the time after radiotherapy. However, goodness of fit was markedly worse for both, number of plaques and intima media thickening.

## Discussion

The deleterious effect of radio(chemo)therapy on the carotid arteries is well known [11, 14–16]. Progressive carotid stenosis was observed over several years after radiotherapy [5, 10, 17], with the same observation being made for the general plaque burden [18, 19]. With regards to IMT, long-term progression was seen in some but not all studies [19–22]. Most of these results, however, are based on cross-sectional studies. Few longitudinal studies include measurements before RT and those have a maximal follow-up of 2 years [8, 12]. While a continuous progression was observed in one study [12], no change could be observed in another one [8]. The present analysis with a maximal follow-up of 4.5 years supports an initial increase in intima media thickness, though no major development could be observed after about one year (Fig. 1 (b)).

Whether RT causes the formation of new plaques or accelerates existing atherosclerosis is important for developing a risk assessment for different patients and potentially employing countermeasures. The present study confirmed the formation of new plaques, which has already been observed immediately after RT [23]. Our results do not show any further increase in the number of plaques about one year after RT (Fig. 1 (a)).

In patients with diabetes mellitus, RT induced thickening of intima media was observed to be less pronounced than in other patients. However, this might have been caused by issues with the retrospective study set-up, and in particular with the small study size. Another weakness of the study design is that imaging was performed for tumor follow-up. As a result, only a fraction of patients with hypopharyngeal carcinoma could be included. Due to the limited resolution of the CT scans, small plaques might have been overlooked and respective volume measurements remain difficult. Although these limitations decrease the statistical power of the results, they are unlikely to yield a bias: scans were performed in a standardized way and image analysis was conducted blindly for patient and treatment characteristics. While being conceptually interesting, the number of plaques is problematic for long-term studies as not only new plaques may form, but established plaques may merge. This might have contributed to the apparent decrease in the number of plaques after 1.5 years in Fig. 1 (a), which, however, was not statistically significant, anyway. Finally, it should be noted that a normal progression of atherosclerosis could safely be neglected in this study as typical thickening of the intima media amounts to about 0.01 mm per year [24].

## Conclusion

Radiotherapy of hypopharyngeal carcinoma leads to a persistent thickening of the intima media and formation of new plaques in the carotids. One year after radiotherapy these processes were largely completed, although longer term follow up is warranted. Larger study groups, however, are necessary to confirm this result.

## Supplementary information

**Additional file 1.** Data analysed.

## Data Availability

All data generated or analysed during this study are included in this published article and its supplementary information files.

## Abbreviations

CCA: Common carotid artery
CT: Computed tomography
ICA: Internal carotid artery
IQR: Interquartile range
MRI: Magnetic resonance imaging
RT: Radiotherapy

## References

[1] Plummer C, Henderson RD, O'Sullivan JD, Read SJ (2011). Ischemic stroke and transient ischemic attack after head and neck radiotherapy: a review. Stroke.

[2] Vallerio P, Sarno L, Stucchi M, Musca F, Casadei F, Maloberti A, Lestuzzi C, Mancia G, Moreo A, Palazzi M, Giannattasio C (2016). Long-term effects of radiotherapy on arterial stiffness in breast Cancer women. Am J Cardiol.

[3] Feehs RS, McGuirt WF, Bond MG, Strickland HL, Craven TE, Hiltbrand JB (1991). Irradiation. A significant risk factor for carotid atherosclerosis. Arch Otolaryngol Head Neck Surg.

[4] Dubec JJ, Munk PL, Tsang V, Lee MJ, Janzen DL, Buckley J, Seal M, Taylor D (1998). Carotid artery stenosis in patients who have undergone radiation therapy for head and neck malignancy. Br J Radiol.

[5] Cheng SW, Wu LL, Ting AC, Lau H, Lam LK, Wei WI (1999). Irradiation-induced extracranial carotid stenosis in patients with head and neck malignancies. Am J Surg.

[6] Anzidei M, Suri JS, Saba L, Sanfilippo R, Laddeo G, Montisci R, Piga M, Argiolas GM, Raz E (2016). Longitudinal assessment of carotid atherosclerosis after radiation therapy using computed tomography: a case control study. Eur Radiol.

[7] Martin JD, Buckley AR, Graeb D, Walman B, Salvian A, Hay JH (2005). Carotid artery stenosis in asymptomatic patients who have received unilateral head-and-neck irradiation. Int J Radiat Oncol Biol Phys.

[8] Kim BJ, Kang HG, Lee SW, Jung J, Lee MH, Kang DW, Kim JS, Kwon SU (2018). Changes in the common carotid artery after radiotherapy: wall thickness, calcification, and atherosclerosis. J Clin Neurol.

[9] Wilbers J, Meijer FJ, Kappelle AC, Kaanders JH, Boogerd W, Dorresteijn LD, van Dijk EJ, Steens SC (2015). Magnetic resonance imaging of the carotid artery in long-term head and neck cancer survivors treated with radiotherapy. Acta Oncol.

[10] Zhou L, Xing P, Chen Y, Xu X, Shen J, Lu X (2015). Carotid and vertebral artery stenosis evaluated by contrast-enhanced MR angiography in nasopharyngeal carcinoma patients after radiotherapy: a prospective cohort study. Br J Radiol.

[11] Bashar K, Healy D, Clarke-Moloney M, Burke P, Kavanagh E, Walsh SR (2014). Effects of neck radiation therapy on extra-cranial carotid arteries atherosclerosis disease prevalence: systematic review and a meta-analysis. PLoS One.

[12] Muzaffar K, Collins SL, Labropoulos N, Baker WH (2000). A prospective study of the effects of irradiation on the carotid artery. Laryngoscope.

[13] Walker LJ, Ismail A, McMeekin W, Lambert D, Mendelow AD, Birchall D (2002). Computed tomography angiography for the evaluation of carotid atherosclerotic plaque: correlation with histopathology of endarterectomy specimens. Stroke.

[14] Abayomi OK (2004). Neck irradiation, carotid injury and its consequences. Oral Oncol.

[15] Gujral DM, Chahal N, Senior R, Harrington KJ, Nutting CM (2014). Radiation-induced carotid artery atherosclerosis. Radiother Oncol.

[16] Xu J, Cao Y (2014). Radiation-induced carotid artery stenosis: a comprehensive review of the literature. Interv Neurol.

[17] Cheng SW, Ting AC, Ho P, Wu LL (2004). Accelerated progression of carotid stenosis in patients with previous external neck irradiation. J Vasc Surg.

[18] Chang YJ, Chang TC, Lee TH, Ryu SJ (2009). Predictors of carotid artery stenosis after radiotherapy for head and neck cancers. J Vasc Surg.

[19] Yuan C, Wu VW, Yip SP, Kwong DL, Ying M (2014). Predictors of the extent of carotid atherosclerosis in patients treated with radiotherapy for nasopharyngeal carcinoma. PLoS One.

[20] Gujral DM, Shah BN, Chahal NS, Bhattacharyya S, Hooper J, Senior R, Harrington KJ, Nutting CM (2016). Carotid intima-medial thickness as a marker of radiation-induced carotid atherosclerosis. Radiother Oncol.

[21] Dorresteijn LD, Kappelle AC, Scholz NM, Munneke M, Scholma JT, Balm AJ, Bartelink H, Boogerd W (2005). Increased carotid wall thickening after radiotherapy on the neck. Eur J Cancer.

[22] Huang TL, Hsu HC, Chen HC, Lin HC, Chien CY, Fang FM, Huang CC, Chang HW, Chang WN, Huang CR, Tsai NW, Kung CT, Wang HC, Lin WC, Cheng BC, Su YJ, Chang YT, Chang CR, Tan TY, Lu CH (2013). Long-term effects on carotid intima-media thickness after radiotherapy in patients with nasopharyngeal carcinoma. Radiat Oncol.

[23] Toprak U, Aytas I, Ustuner E, Habiboglu R, Aslan N, Pasaoglu E, Karademir A (2012). Sonographic assessment of acute changes in plaque size and echogenicity and in intima-media thickness of carotid arteries after neck radiation therapy. J Clin Ultrasound.

[24] Tattersall MC, Gassett A, Korcarz CE, Gepner AD, Kaufman JD, Liu KJ, Astor BC, Sheppard L, Kronmal RA, Stein JH (2014). Predictors of carotid thickness and plaque progression during a decade: the multi-ethnic study of atherosclerosis. Stroke.

